# Evaluation of sexual dysfunction and female sexual dysfunction indicators in women with type 2 diabetes: a systematic review and meta-analysis

**DOI:** 10.1186/s13098-019-0469-z

**Published:** 2019-08-27

**Authors:** Elham Rahmanian, Nader Salari, Masoud Mohammadi, Rostam Jalali

**Affiliations:** Department of Nursing, School of Nursing and Midwifery, Kermanshah University of Medical Sciences, Kermanshah, Iran

**Keywords:** Prevalence, Disorder, Type 2 diabetes, Sexual function, Meta-analysis

## Abstract

Type 2 diabetes is one of the most common chronic diseases worldwide, and one of the long-term complications of this disease is sexual dysfunction in women with type 2 diabetes, which has been studied in fewer studies. The aim of this study is to determine the overall prevalence of sexual dysfunction in women with type 2 diabetes and its indicators with systematic and meta-analysis approach. The present meta-analysis study reviewed articles published foreign journals by searching the MEDLINE, Cochrane Library, Science direct, Embase, Proquest and Persian databases, including Iranmedex, Magiran, and SID between January 2000 to December 2018. The heterogeneity of studies was studied using the I^2^ index and data analysis was carried out in Comprehensive Meta-Analysis software. The Meta-analysis review of 25 studies and 3892 individuals aged 70–18 years showed that the overall prevalence of sexual dysfunction in women with type 2 diabetes was 68.6% (95% CI 61.1–75.3%). The highest and lowest prevalence of sexual dysfunction was 94.4% in Iranian women with type 2 diabetes (95% CI 91.9%–96.3%) in 2014 and 17% in Italian women with diabetes Type 2 (95% CI 6.4–36.9%) in 2015. Results of meta-regression showed that with the increase in sample size and year of study, the overall prevalence of sexual dysfunction decreased and increased, respectively and the differences were statistically significant (P < 0.05). Regarding the high prevalence of sexual dysfunction in women with type 2 diabetes, health policymakers need to take appropriate measures to address this disorder in patients with type 2 diabetes.

## Background

Diabetes mellitus is characterized by hyperglycemia and carbohydrate metabolism, fat, and protein deficiency. It is one of the most common and costly chronic diseases in the world, and affects about 2–5% of adults in industrial societies [1]. Given the increasing number of diabetes in the world, the World Health Organization (WHO) has described it as a hidden epidemic, with a global prevalence of 6.4% (285 million people) among 20–79 age group in 2010. However, this figure is projected to increase to 7.7% in 2030 (439 million per year), and to more than 600 million per year in 2035 [2]. There are now more than 380 million people with diabetes worldwide, and this figure is estimated to be 29.1 million people in the United States, that is 9.3% of the total population [3]. The statistics provided by the International Federation of Diabetes show that approximately 5 million people aged 20–79 years are suffering from diabetes in Iran, with a prevalence of 8.5% [4]. Diabetes is known as one of the most important risk factors for cardiovascular disease, with diabetic patients being at risk of cardiovascular disease 2 to 4 times higher than the general population [2–4]. Diabetes can manifest as microvascular or macrovascular complications. The most important microvascular complications include nephropathy, retinopathy, neuropathy and the most important macrovascular complications include hypertension and coronary artery disease [1–4]. This chronic disease is also able to cause severe ocular, renal, and neuropathic complications in patients to blindness, renal, cardiovascular disease, amputation, and disability [5–7]. Such chronic and debilitating conditions have widespread effect on the ability of the individual and the daily activities of these patients, which in turn affects the quality of life in these patients [8].

One of the long-term complications of diabetes is sexual dysfunction. Previous studies have shown the negative effects of this disease on the sexual function and health of people with diabetes in both genders [9–11]. Sexual health is defined by WHO as a physical, emotional, psychological, and social well-being in terms of sexual desire, not just lack of disease, dysfunction, or disability [12], regardless of the range of sexual desires that are the basis of important behaviors and lead to sexual health. Sexual health is the result of interaction of cardiovascular, neurological, and hormonal factors and is influenced by individual factors, interpersonal relationships, traditions governing family and community, culture, and religion [13, 14]. Sexual dysfunction is a heterogeneous combination of disorders that is characterized as a major disorder in one’s ability to respond to sexual response or sexual pleasure. These disorders include abnormalities in women’s orgasm, arousal, pain, and unknown sexual dysfunction [15]. Although various studies have reported high prevalence of sexual dysfunction in women with diabetes compared with non-diabetic women [16–31], the sexual problems of diabetic women and its related risk factors are not well defined and highlighted. There are also fewer studies on diabetic women than diabetic men [32–37]. In a study conducted in Iran, the prevalence of sexual dysfunction was reported to be 88% in women with type 2 diabetes [22], which can significantly affect their interest, satisfaction, and ability to participate in sexual activity. Such conditions can be due to vascular, neurological, and psychological problems caused by diabetes or the result of negative effects of the drugs used in these patients [32–38]. On the one hand, the sexual health of diabetic patients, as a component of care, has often been neglected, perhaps because it has been considered by many individuals as a taboo and has thus been neglected [39]. On the other hand, previous studies provide non-transparent and different information and various studies show in consistent scores for sexual dysfunction indices in women with type 2 diabetes. Thus, the aim of this systematic review and meta-analysis is to answer the questions, what is the level of overall prevalence of sexual dysfunction in women with type 2 diabetes and the overall score of female sexual function index in women with diabetes?

## Methods

### Search strategy

This study is a systematic review and meta-analysis and is the result of findings of previous relevant researches. First, articles published in domestic and foreign journals were retrieved by searching MEDLINE, Cochrane Library, Sciencedirect, Embase, Proquest databases and Persian databases, including Iranmedex, Magiran, and SID were restored during January 2000 to December 2018. The researcher carried out the searching process by using the keywords of Diabetes, Non-insulin dependent diabetes, Type 2 diabetes, Sexual dysfunction, and Women in Persian sources, and Diabetes, Non-insulin-dependent diabetes, Sexual dysfunction, women in English databases, as well as both Persian and English words in the Google scholar search engine. It should be noted that (AND) and (OR) operators were combined in order to provide more comprehensive access to all articles; therefore the OR operators was used to check the common names for a disorder like (Blood glucose OR Hyperglycemia), (Diabetes OR Non-insulin dependent diabetes), (Sexual Behavior OR Sexual Orientation), (Disorders OR Dysfunction). AND operator was also used between the keywords (Diabetes AND Non-Insulin-Dependent Diabetes AND Sexual Dysfunction) through the matching words in the MeSH browser.

### Criteria for selection and evaluation of articles

First, all the articles were collected using the selected keywords and when the search was completed, a list of abstracts was prepared and after hiding the articles’ profile, including the name of the journal and the author, the full text of the articles was provided to the reviewers. Each article is read independently by two reviewers. If the article is rejected, the reason was mentioned. If there is a difference between two reviewers, the article was judged by the third reviewer and the third referee’s opinion was taken into account. Inclusion criteria included Persian and English articles based on cross-sectional, case–control, and cohort studies to obtain the prevalence of sexual dysfunction in women with type 2 diabetes as well as to investigate the relationship between clinical and metabolic factors with sexual dysfunction in women with type 2 diabetes. Review, interventional articles, as well as articles with no access to their full text or poor rating quality, were excluded from the list of final articles. Moreover, studies that reported female sexual function indices (FSFI), including desire, arousal, lubrication, orgasm, satisfaction, and pain in women with type 2 diabetes were also separated from the final studies so that they will be used in the meta-analysis phase while investigating mean score and overall standard deviation of these indices. In order to investigate gray literature, that part of the evidence and documentation that has not been published for any reason, a comprehensive review on Google has been put on the agenda in the present study. To review the articles collected, a checklist of articles’ profile, included the researcher’s name, the article’s title, the year and place of the study, the sample size, the prevalence of the disorder in studies based on the PRISMA chart, included a four-step process consisting of initial search and identification of the studies based on keywords, screening of articles based on duplicate searches and similar articles, the criteria for accepting articles based on inclusion and exclusion criteria, and finally the articles selected for entering meta-analysis, was prepared. Duplicate and multiple publications from the same population will be removed using citation management, software EndNote (version X7, for Windows, Thomson Reuters).

### Quality assessment

STROBE checklist was used to investigate the studies. This checklist consists of 22 sections, 18 of which are general and are used for all observational studies, including cohort, case–control, and cross-sectional. There are also 4 specific sections that depend on the type of study, and various methodological aspects that include objectives of the study, determining the appropriate sample size, type of study, sampling method, research population, data collection method, variables definition, and method, data collection tools, objectives under study, statistical test, and findings of the study. Accordingly, the maximum score is considered as 32, and articles with a score > 14 were considered to be low in terms of quality assessment and excluded from the study.

### Statistical analysis

Heterogeneity was investigated using the I^2^ test. Generally, heterogeneity is classified into three categories, heterogeneity > 25% (low heterogeneity), 25–75% (average heterogeneity), and 75% > higher (high heterogeneity). Data analysis was carried out using Comprehensive Meta-analysis software (Biostat, Englewood, NJ, USA version 3) and probability bias of the results was calculated by using funnel plot and Egger test (P < 0.05), Begg and Mazumdar (P < 0.1). Moreover, meta-regression test in two dimensions of sample size and years of research was used to investigate the effects of the potentially effective factors on the heterogeneity of the studies.

## Results

### Search output

**Fig. 1:**
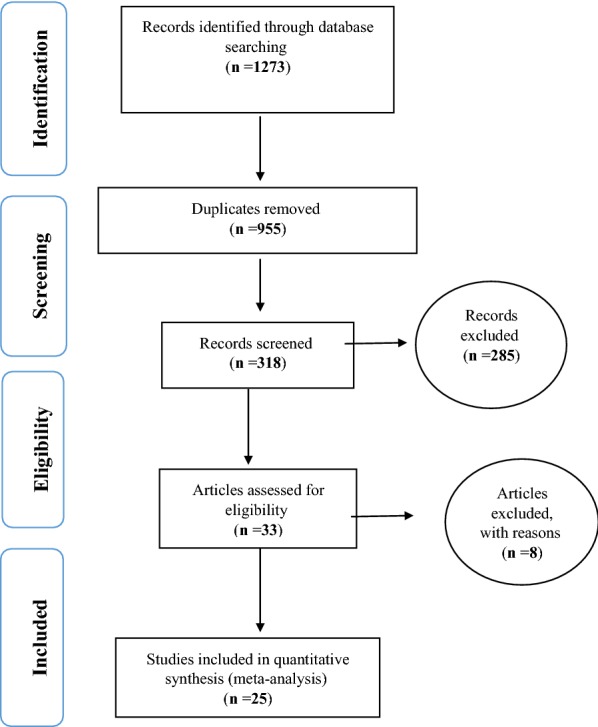
The flowchart on the stages of including the studies in the systematic review and meta-analysis (PRISMA 2009)

Based on studies on the prevalence of sexual dysfunction in women with type 2 diabetes, including articles published in domestic and foreign journals, and searching Iranmedex, Magiran, and SID databases (n = 89 articles), Medline (PubMed) (n = 112 articles), and Sciencedirect (n = 583 articles), Proquest (n = 63 articles), Embase, (n = 52 articles), Cochrane Library (n = 12 articles), and Google Scholar search engines(n = 366 articles), and a total of 1273 articles were obtained. Then, a total of 955 duplicate articles were excluded and 318 articles met the inclusion criteria based on preliminary studies. Finally, 25 articles were entered the meta-analysis phase after eliminating 285 unrelated articles and 8 articles with no access to their abstracts and original articles as well as their low quality during the secondary investigation (Fig. 1). Profiles of these articles were reported in Table 1 [31, 34, 40–62].

**Table 1:** Specifications of studies entered the study

Row	Author [references]	Publication year	Area	Participants’ age	Sample size	Prevalence	Quality assessment
1	Erol et al. [40]	2003	Turkey	38.8 ± 1.1	72	51.3	Moderate
2	Doruk et al. [41]	2005	Turkey	41 ± 1.1	50	42	Moderate
3	Najafi et al. [42]	2006	Iran	48.1 ± 9.5	26	76	Moderate
4	Mezones et al. [43]	2008	Peru	53.3 ± 4.1	36	75	Moderate
5	Ogbera et al. [44]	2009	Nigeria	50.5 ± 1.2	58	87.9	Moderate
6	Esposito et al. [45]	2010	Italy	57.9 ± 6.9	595	53.4	Moderate
7	Ziaeirad et al. [46]	2010	Iran	46.3 ± 5.2	91	86.8	High
8	Yencilk et al. [34]	2010	Turkey	39.2 ± 9.4	62	80.6	Moderate
9	Giugliano et al. [47]	2010	Italy	35–70	659	53.1	Moderate
10	Nowosielski et al. [48]	2011	Poland	47.6 ± 7.02	133	27.06	Moderate
11	Shi et al. [49]	2012	China	47.3 ± 7.1	106	79.2	High
12	Sharifiaghdas et al. [50]	2012	Iran	42.1 ± 5.9	45	71	Moderate
13	Cortelazzi et al. [31]	2013	Italy	18–50	34	67.6	Moderate
14	Erten et al. [51]	2013	Turkey	51.3 ± 5.8	38	47.4	Moderate
15	Vafaeimanesh et al. [52]	2014	Iran	48.2 ± 6.6	110	53.6	Moderate
16	Cordero et al. [53]	2014	Mexico	40–70	120	81.6	Moderate
17	Shadman et al. [54]	2014	Iran	54.4 ± 9.8	420	94.4	Moderate
18	Bjerggaard te al [55]	2015	Denmark	65 ± 7	348	68.3	High
19	Elyasi et al. [56]	2015	Iran	42 ± 10.1	150	78.7	Moderate
20	Mazzilli et al. [57]	2015	Italy	36.6 ± 5.2	24	17	Moderate
21	Ammar et al. [58]	2016	Tunisia	40.8 ± 4.8	30	50	Moderate
22	Li et al. [59]	2016	China	51 ± 9	184	75	Moderate
23	Bak et al. [60]	2017	Poland	51.1 ± 9.7	114	68	Moderate
24	Turki et al. [61]	2017	Saudi Arabia	40–50	275	88.7	Moderate
25	Marques et al. [62]	2018	Portugal	45–70	112	70.9	Moderate

### Heterogeneity and publication bias

**Fig. 2:**
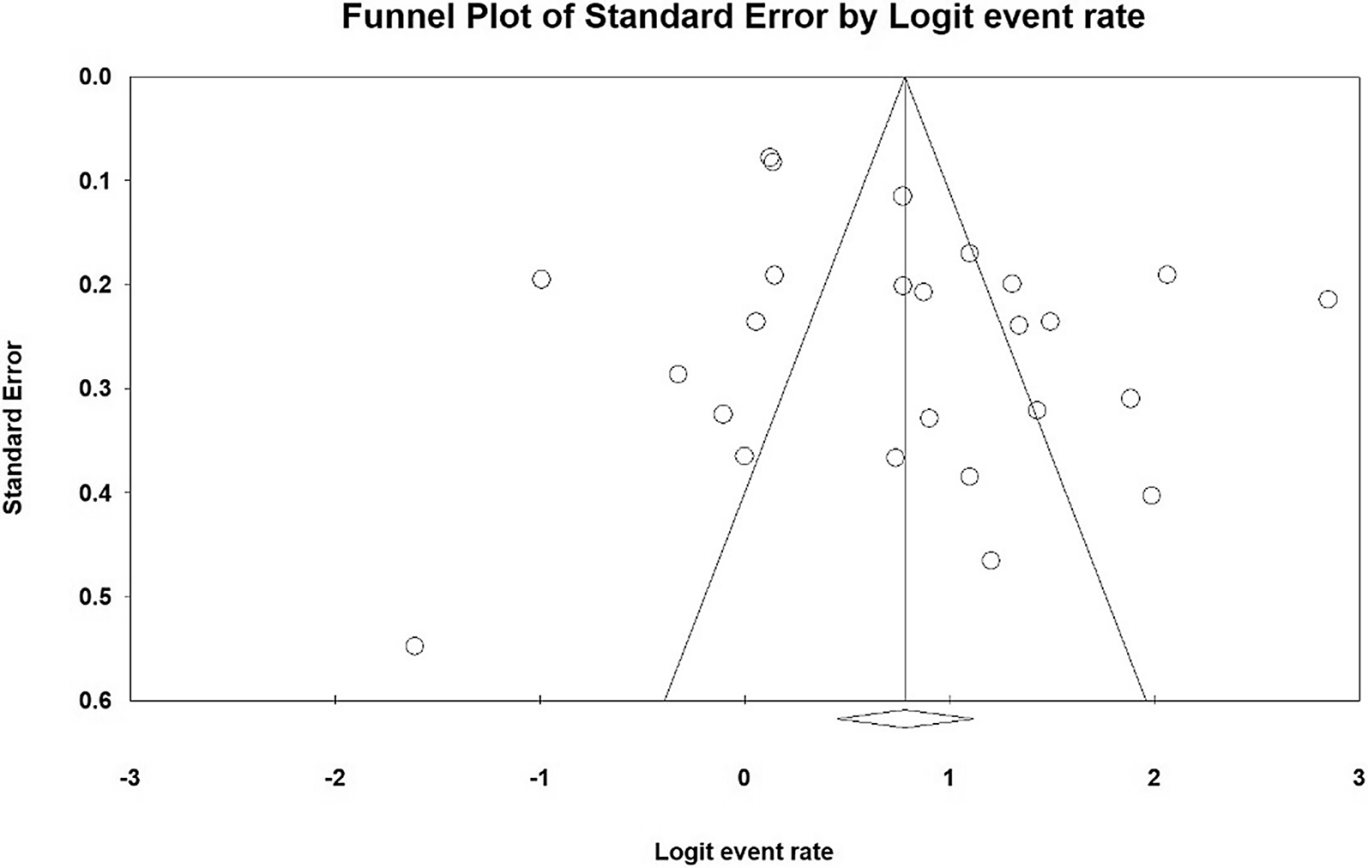
Results of funnel plot on the prevalence of sexual dysfunction in women with type 2 diabetes

The heterogeneity of the studies was evaluated using the I^2^ test and its value was obtained I^2^ = 94.5% indicating a high heterogeneity in the studies included. Therefore, the random effects model was used to combine the results of the studies. The results of the publication bias were measured using Egger test (Fig. 2), which was not statistically significant (P = 0.103), Begg and Manzumdar test also showed no significant publication bias in the present study (P = 0.833).

**Fig. 3:**
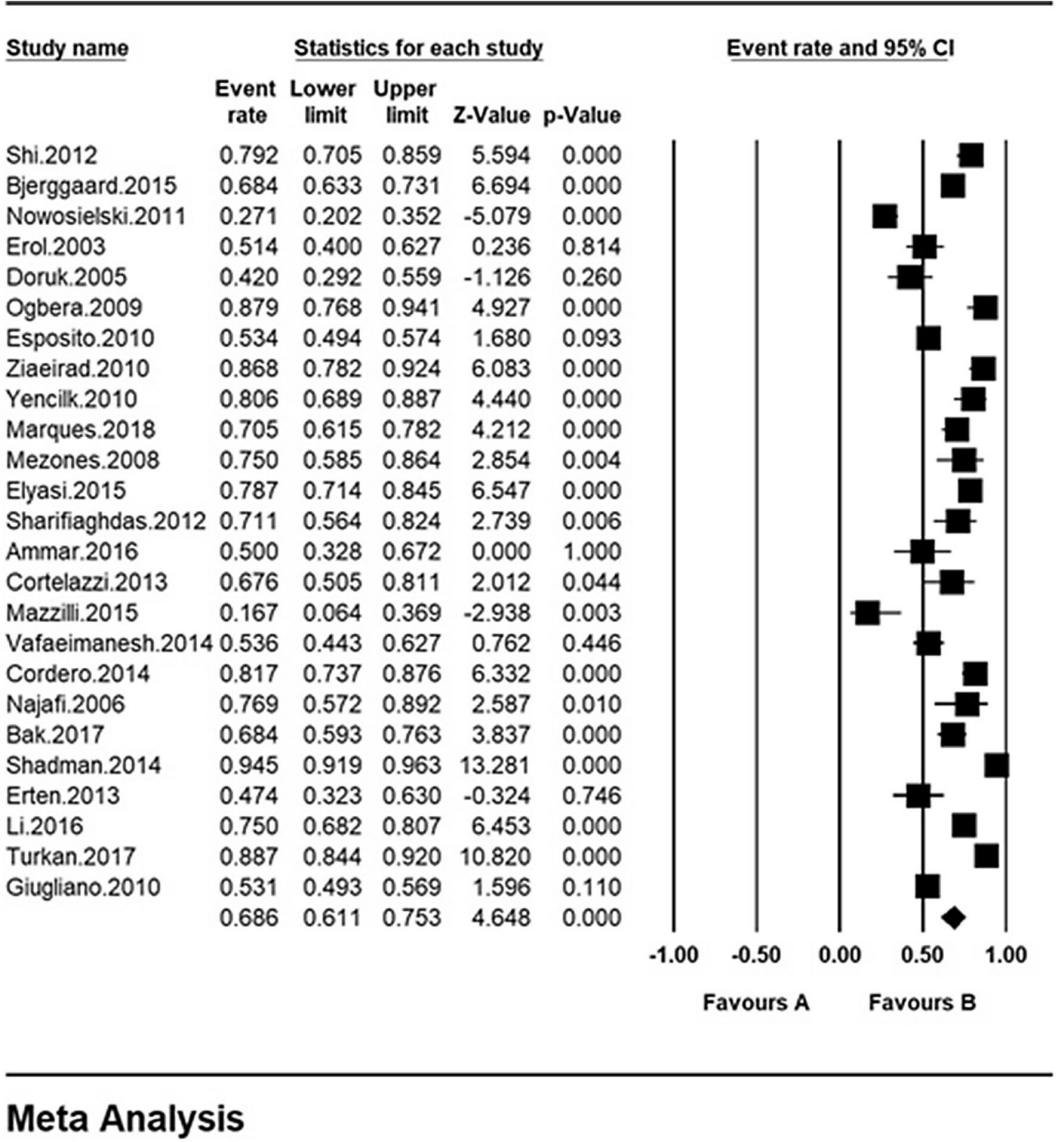
Overall prevalence of sexual dysfunction in women with type 2 diabetes based on the random model

The total sample size was 3892 with the age range of 18–70 years. The overall prevalence of sexual dysfunction in women with type 2 diabetes was 68.6% based on the meta-analysis (95% CI 61.1–75.3%). The highest prevalence of sexual dysfunction was 94.4% among Iranian women with type 2 diabetes (95% CI 91.9%–96.3%) in 2014 [58] and the lowest prevalence was observed among Italian women with type 2 diabetes 17% (95% CI 6.4–36.9%) in 2015 [53] (Fig. 3). In this figure, the prevalence of sexual dysfunction is shown based on the random effects model where the black square indicates the prevalence rate and the length of the line segment on which is located shows the 95% CI interval in each study, the diamond sign also shows the prevalence rate in all studies.

### Sensitivity analysis

**Fig. 4:**
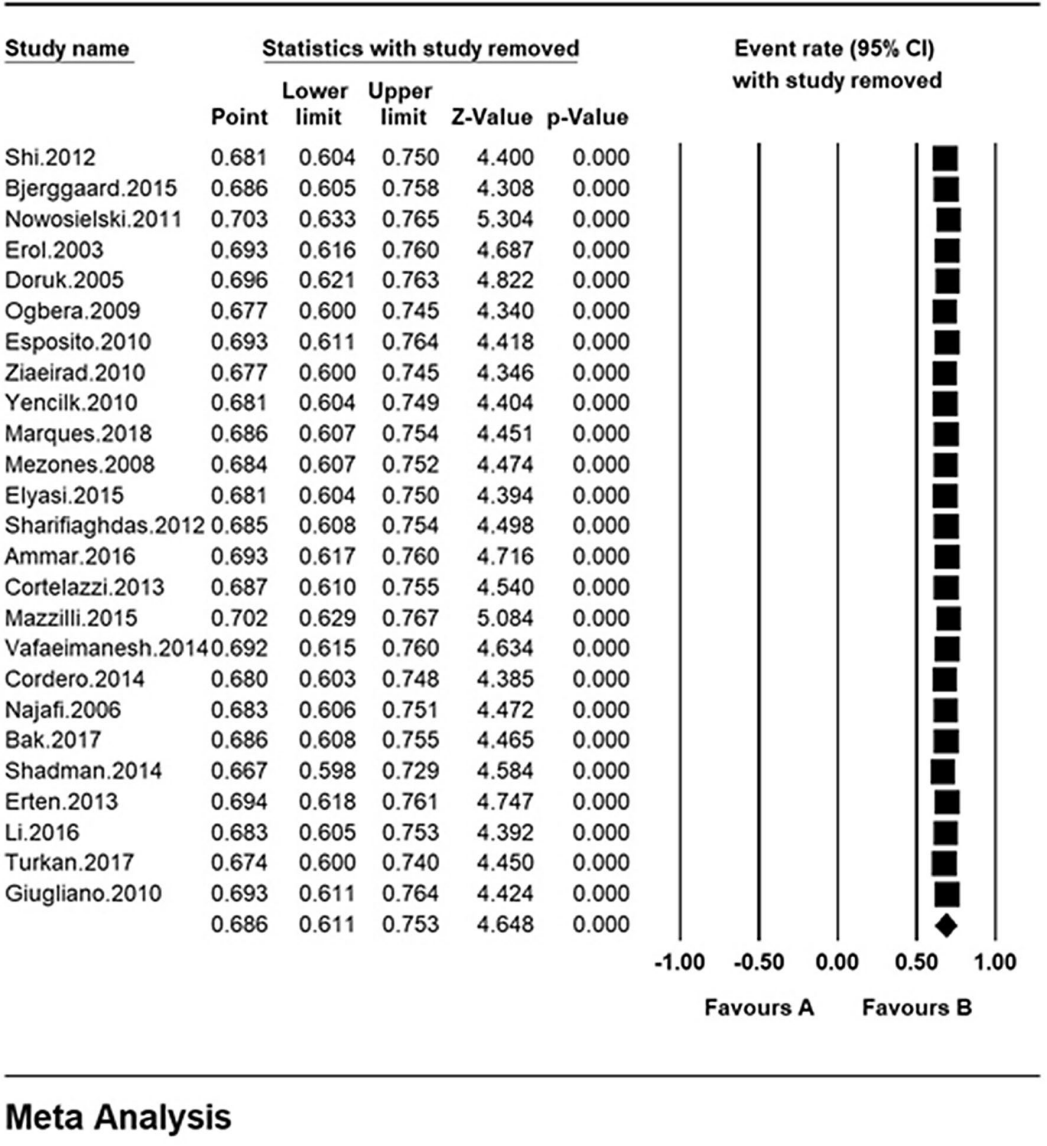
Results of sensitivity analysis

A sensitivity analysis was performed to ensure the stability results, after removing each study results did not change (Fig. 4).

### Meta-regression analysis

**Fig. 5:**
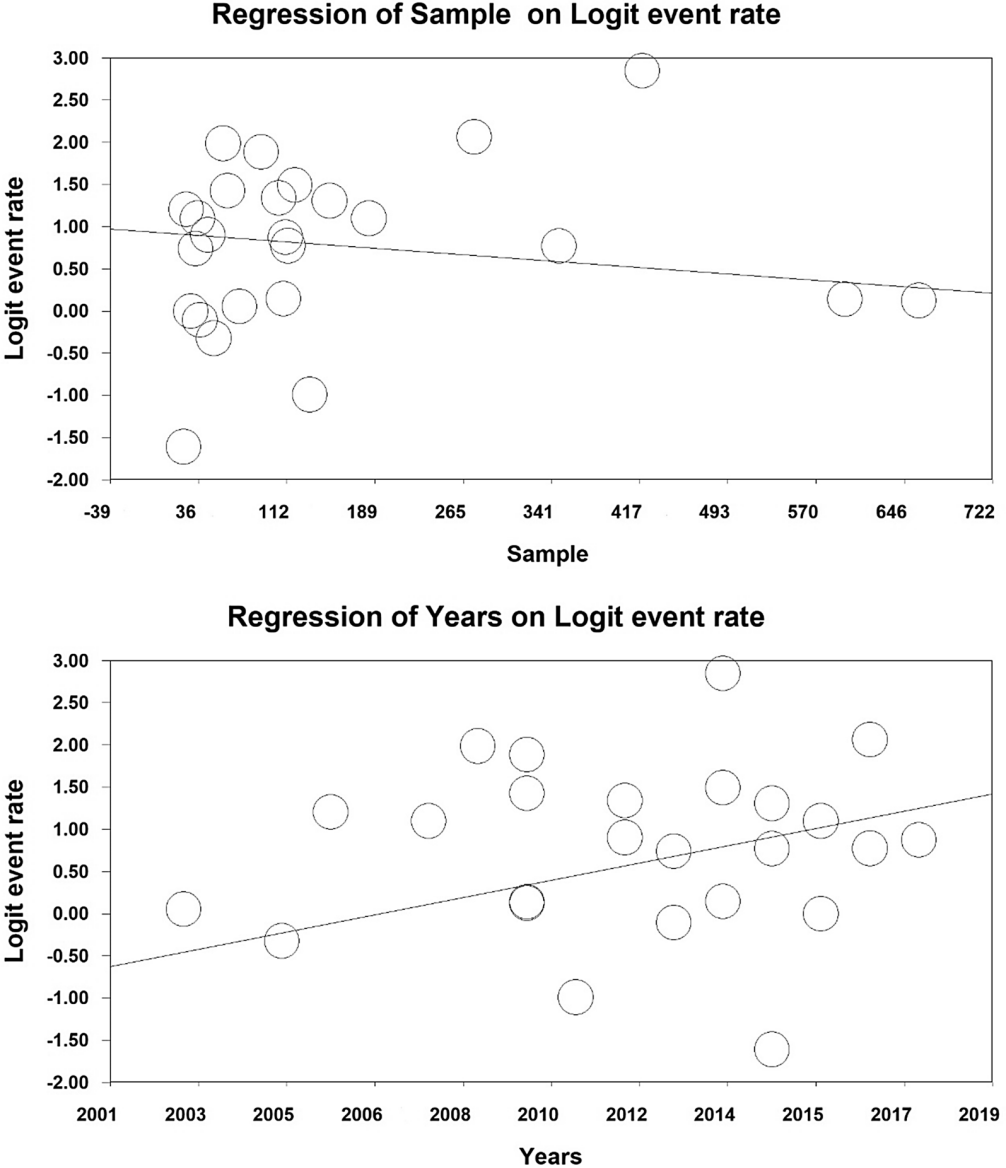
Meta-regression diagram of the prevalence of sexual dysfunction in women with type 2 diabetes by sample size and year of publication

In order to investigate the effects of potentially effective factors on the heterogeneity of the prevalence of sexual dysfunction in women with type 2 diabetes, meta-regression test was applied to two factors of sample size and year of study (Fig. 5). With an increase in the sample size, the overall prevalence of sexual dysfunction is reduced in women with Type 2 diabetes, which is statistically significant (Fig. 4) (P < 0.05). It is also reported in this figure that with an increase in the year of the research, the overall prevalence of sexual dysfunction in women with type 2 diabetes is increased, which was also statistically significant (P < 0.05).

### Scores of female sexual function index

In this study, information on FSFI score including desire, arousal, lubrication, orgasm, satisfaction, and pain were also extracted from the studies reviewed. The mean score in women of the case group (type II diabetes) was lower than that of the control group in all studies that examined these indices, and these values were statistically significant in all studies (Table 2) (P < 0.05).

**Table 2:** Specifications of studies entered the study (Female Sexual Function Index)

Female Sexual Function Index	Studies [references]	N	Case (mean ± SD)	N	Control (mean ± SD)	P value
Desire	Shi et al. [49]	106	2.43 ± 0.99	100	3.04 ± 1.04	0.000
	Nowosielski et al. [48]	133	3.45 ± 1.24	109	3.91 ± 1.13	0.000
	Mezones et al. [43]	36	2.6 ± 1.4	36	3.8 ± 1.1	0.000
	Ogbera et al. [44]	58	2.3 ± 0.9	38	3 ± 0.6	0.000
	Sharifiaghdas et al. [50]	45	32 ± 1.1	90	47 ± 56.5	0.000
	Afshari et al. [75]	106	3.34 ± 1.37	128	3.69 ± 1.19	0.000
	Ammar et al. [58]	30	2.86 ± 1.2	30	4 ± 1	0.000
	Mazzilli et al. [57]	24	5.4 ± 1.2	45	6.8 ± 1.7	0.000
	Bak et al. [60]	101	6.8 ± 3.03	89	8.9 ± 1.08	0.000
	Li et al. [59]	184	2.38 ± 1.06	146	2.82 ± 1.17	0.000
Arousal	Shi et al. [49]	106	2.54 ± 1.5	100	3.26 ± 1.31	0.000
	Nowosielski et al. [48]	133	3.75 ± 1.76	109	4.4 ± 1.35	0.000
	Yencilk et al. [34]	62	3.09 ± 1.81	50	5.47 ± 1.95	0.000
	Mezones et al. [43]	36	3.5 ± 1.9	36	4.7 ± 0.8	0.000
	Veronelli et al. [76]	28	3.4 ± 0.39	36	5 ± 0.16	0.000
	Ogbera et al. [44]	58	2 ± 1.5	38	3.2 ± 1.5	0.000
	Sharifiaghdas et al. [50]	45	38 ± 84.4	90	61 ± 67	0.000
	Afshari et al. [75]	106	3.66 ± 1.37	128	3.79 ± 1.39	0.000
	Ammar et al. [58]	30	3.13 ± 0.7	30	4.3 ± 0.8	0.000
	Cortelazzi et al. [31]	48	4.1 ± 0.16	61	4.5 ± 0.11	0.000
	Naghavi et al. [77]	50	22.8 ± 0.5	40	15.2 ± 0.1	0.000
	Li et al. [59]	184	2.47 ± 1.53	146	3.07 ± 1.42	0.000
Lubrication	Shi et al. [49]	106	3.25 ± 1.89	100	4.22 ± 1.72	0.000
	Nowosielski et al. [48]	133	4.53 ± 1.72	109	5.01 ± 1.45	0.000
	Esposito et al. [45]	322	2.5 ± 0.9	273	3.4 ± 1	0.000
	Yencilk et al. [34]	62	4.31 ± 2.2	50	5.68 ± 1.19	0.000
	Olarinoye et al. [78]	52	2.51 ± 2.07	39	5 ± 1.91	0.000
	Erol et al. [40]	72	4.3 ± 1	60	4.6 ± 0.6	0.000
	Mezones et al. [43]	36	3.2 ± 1.9	36	4.5 ± 1.3	0.000
	Veronelli et al. [76]	28	3.6 ± 0.46	36	5.6 ± 0.11	0.000
	Afshari et al. [75]	106	3.62 ± 1.25	128	4.05 ± 1.39	0.000
	Ammar et al. [58]	30	4.3 ± 0.6	30	5.2 ± 0.5	0.000
	Cortelazzi et al. [31]	48	4.6 ± 0.17	61	5 ± 0.12	0.000
	Naghavi et al. [77]	50	22.4 ± 0.6	40	15.2 ± 0.1	0.000
	Li et al. [59]	184	3.14 ± 1.86	146	3.88 ± 1.68	0.000
Orgasm	Shi et al. [49]	106	2.89 ± 1.76	100	3.76 ± 1.66	0.000
	Nowosielski et al. [48]	133	4.45 ± 1.23	109	4.78 ± 1.21	0.000
	Yencilk et al. [34]	62	3.91 ± 2.7	50	5.01 ± 1.96	0.000
	Olarinoye et al. [78]	52	2.02 ± 1.41	39	3.22 ± 1.38	0.000
	Erol et al. [40]	72	3.5 ± 1	60	4.5 ± 0.5	0.000
	Mezones et al. [43]	36	3.2 ± 1.8	36	4.5 ± 1.1	0.000
	Veronelli et al. [76]	28	3.5 ± 0.45	36	5.4 ± 0.12	0.000
	Afshari et al. [75]	106	3.68 ± 1.42	128	3.86 ± 1.47	0.000
	Ammar et al. [58]	30	4 ± 1.1	30	5.17 ± 0.6	0.000
	Cortelazzi et al. [31]	48	4.5 ± 0.15	61	5 ± 0.11	0.000
	Naghavi et al. [77]	50	25.6 ± 0.3	40	15.1 ± 0.1	0.000
	Bak et al. [60]	101	6.8 ± 3.03	89	8.9 ± 1.08	0.000
	Li et al. [59]	184	2.78 ± 1.78	146	3.48 ± 1.6	0.000
Satisfaction	Shi et al. [49]	106	3.63 ± 1.25	100	4.27 ± 1.33	0.000
	Nowosielski et al. [48]	133	4.59 ± 1.09	109	4.94 ± 1.11	0.000
	Yencilk et al. [34]	62	4.51 ± 1.88	50	5.34 ± 1.64	0.000
	Erol et al. [40]	72	3.4 ± 1	60	4.3 ± 0.6	0.000
	Mezones et al. [43]	36	3.8 ± 1.3	36	4.8 ± 0.9	0.000
	Ogbera et al. [44]	58	2.9 ± 2.2	38	4 ± 1.9	0.000
	Afshari et al. [75]	106	4.01 ± 1.33	128	4.08 ± 1.36	0.000
	Ammar et al. [58]	30	4.13 ± 0.8	30	5.25 ± 1.1	0.000
	Naghavi et al. [77]	50	24.2 ± 0.2	40	13.7 ± 0.1	0.000
	Bak et al. [60]	101	8.1 ± 3.5	89	12.8 ± 1.65	0.000
Pain	Yencilk et al. [34]	62	3.14 ± 1.92	50	4.92 ± 2.01	0.000
	Olarinoye et al. [78]	52	2.43 ± 2.04	39	5 ± 1.87	0.000
	Mezones et al. [43]	36	3.1 ± 1.7	36	4.6 ± 1.3	0.000
	Veronelli et al. [76]	28	3.7 ± 0.49	36	5.3 ± 0.24	0.000
	Sharifiaghdas et al. [50]	45	4 ± 8.9	90	23 ± 25.3	0.000
	Li et al. [59]	184	3.65 ± 1.94	146	4.14 ± 1.77	0.000

**Fig. 6:**
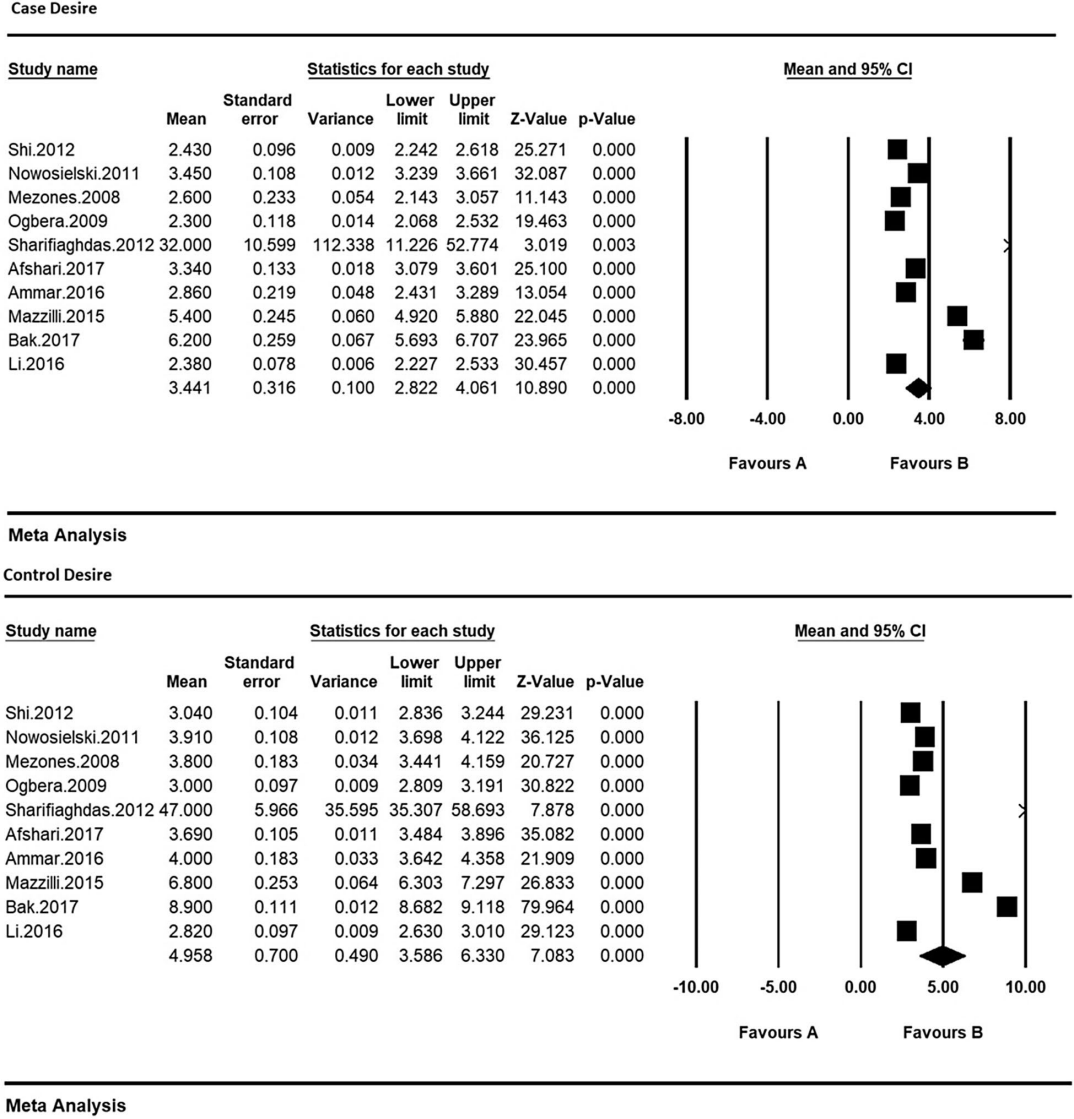
Meta-analysis of mean score of desire index in case and control groups based on the random model

**Fig. 7:**
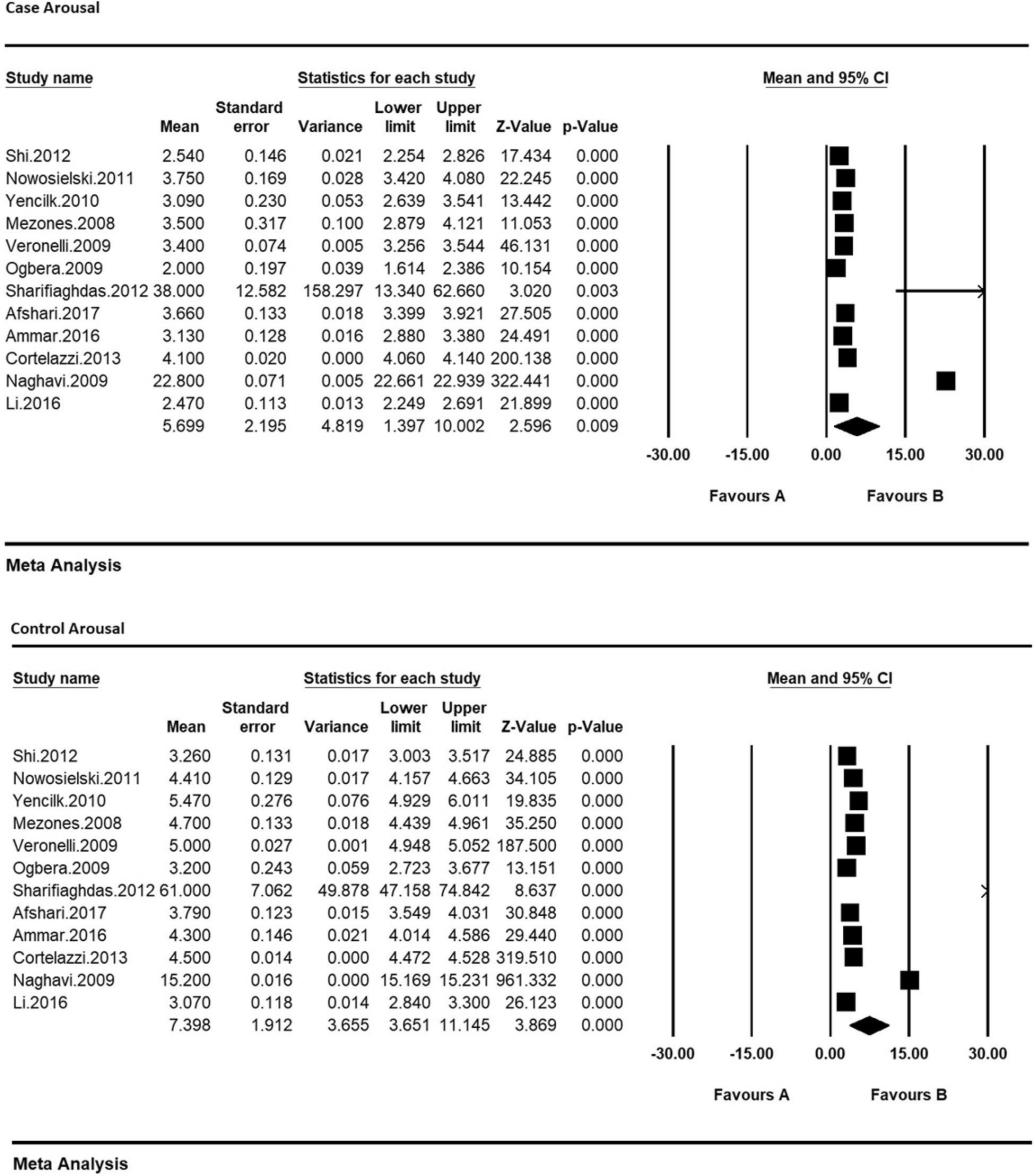
Meta-analysis of the mean score of the arousal index in case and control groups based on the random model

**Fig. 8:**
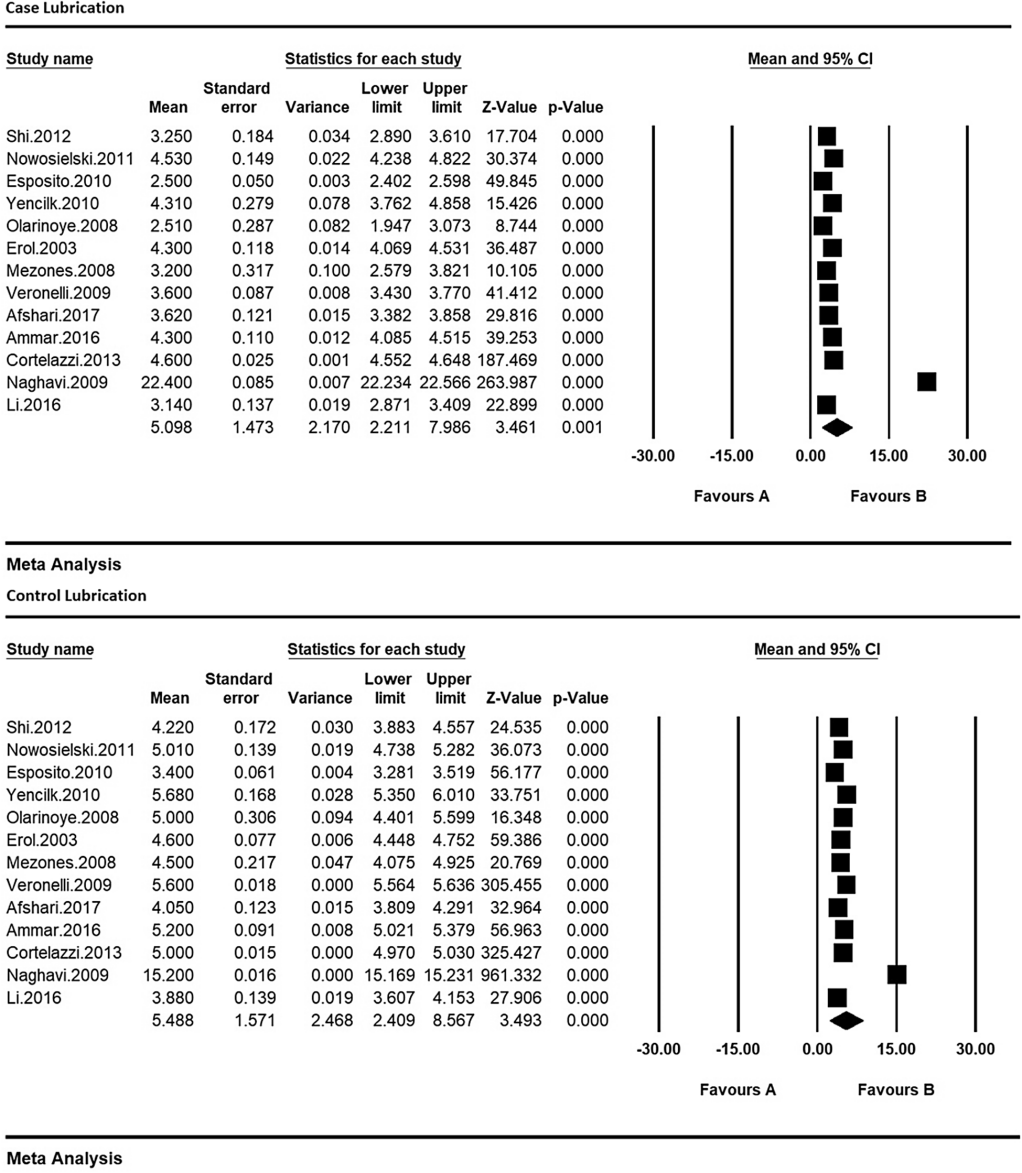
Meta-analysis of the mean score of the lubrication index in case and control groups based on the random model

**Fig. 9:**
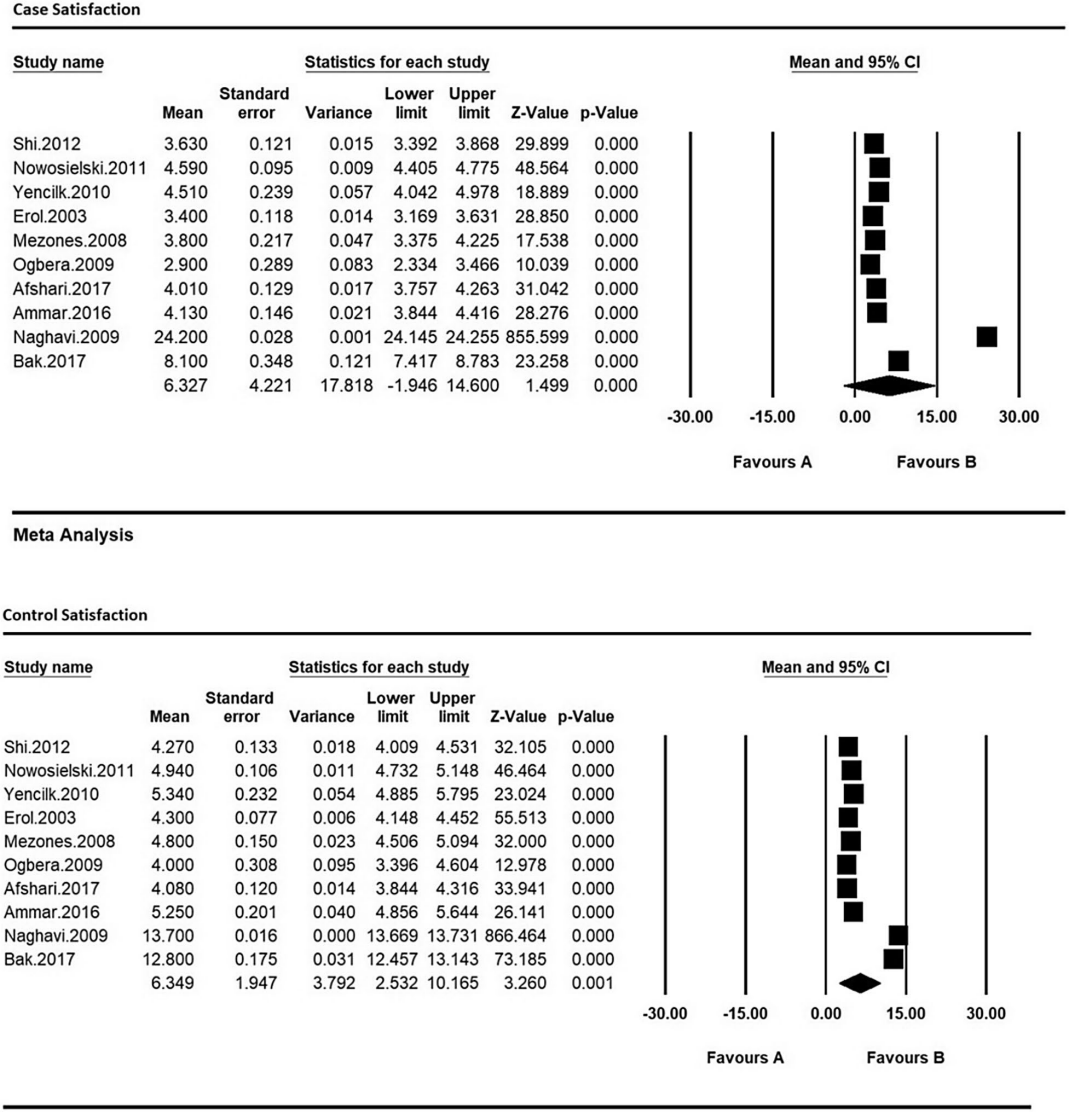
Meta-analysis of mean satisfaction score in case and control groups based on the random model

**Fig. 10:**
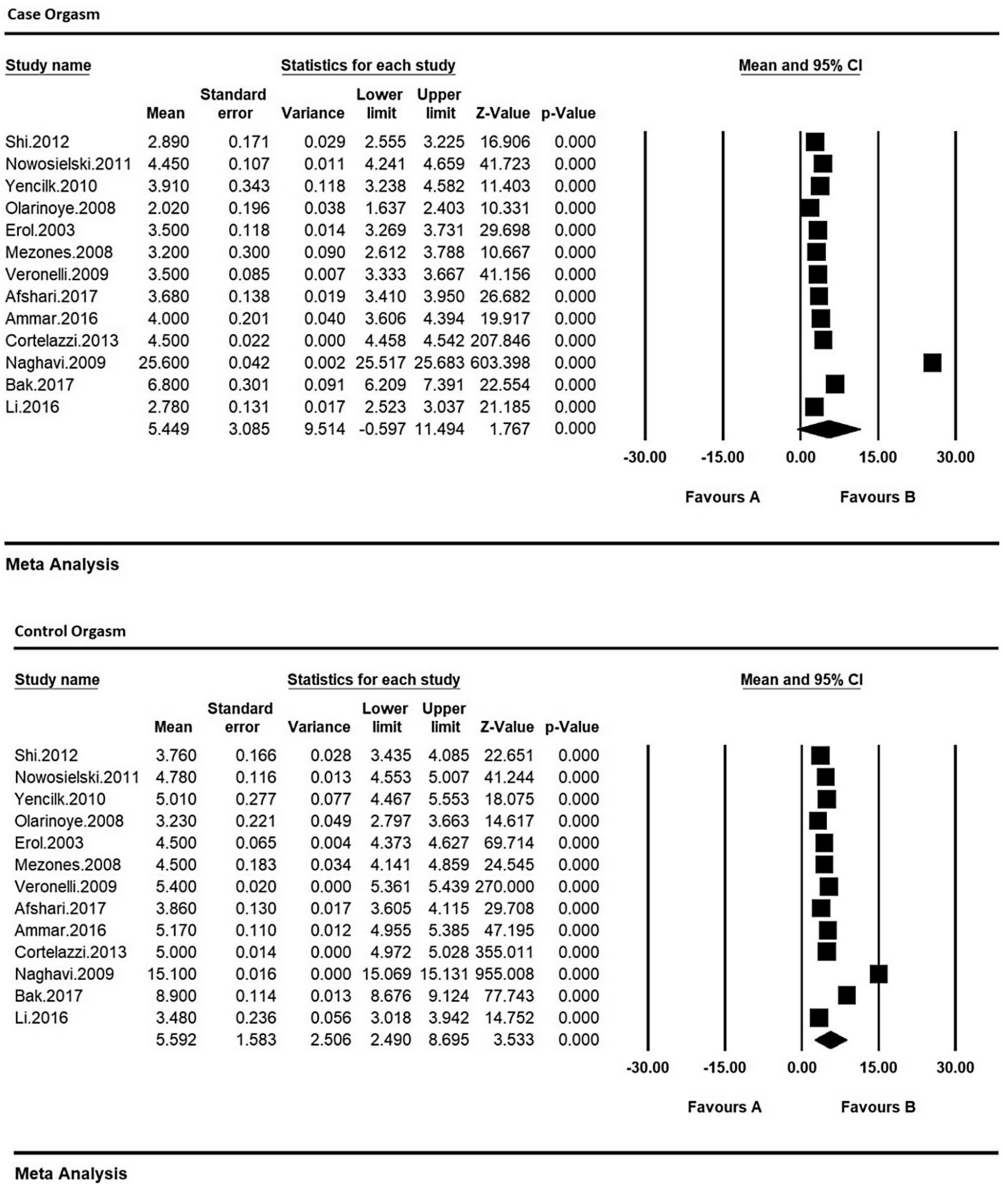
Meta-analysis of the mean orgasm score in case and control groups based on the random model

**Fig. 11:**
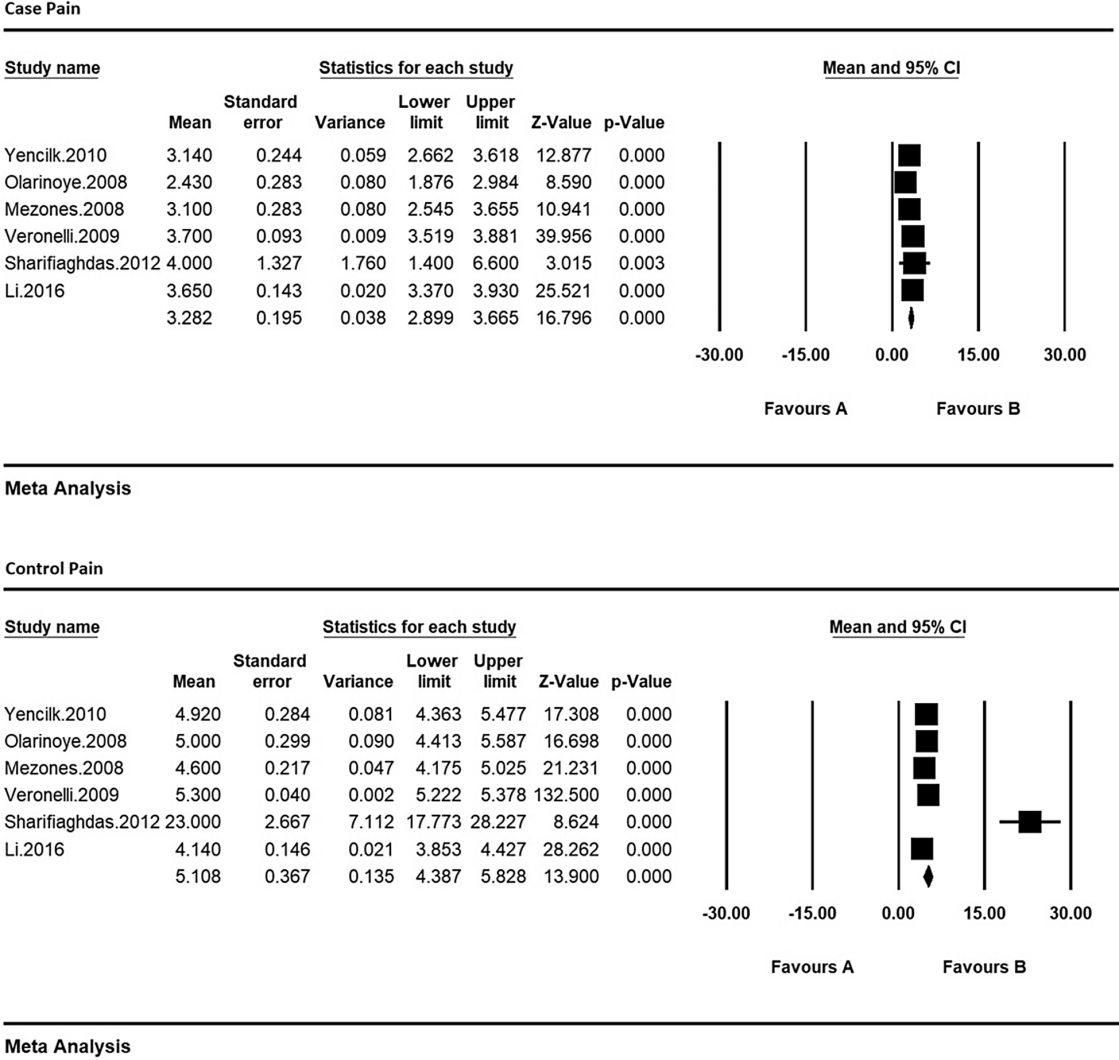
Meta-analysis of the mean pain score in case and control groups based on the random model

Therefore, in order to equalize the values of each of the indices of sexual dysfunction in women with type 2 diabetes and their control group, the meta-analysis was used so as to evaluate the overall mean scores while reviewing all studies. Based on the results of meta-analysis (Figs. 6, 7, 8, 9, 10 and 11), FSFIs scores (expressed as mean ± SD in *t* test analysis) in case and control groups were as follows: Desire, arousal, lubrication, satisfaction, orgasm, and pain in the case and control groups were respectively 3.4 ± 0.31 and 4.9 ± 0.7 (Fig. 6), 5.6 ± 2.1 and 7.3 ± 1.91 (Fig. 7), 5.9 ± 1.47 and 5.4 ± 5.1 (Fig. 8), 6.3 ± 4.2 and 6.3 ± 1.9 (Fig. 9), 5.4 ± 0.08 and 5.5 ± 1.5 (Fig. 10), 3.28 ± 0.19 and 5.1 ± 0.36 (Fig. 11). There was a significant difference between the case and control groups in all studies in terms of FSFI indices and the mean scores were lower in the case group (women with diabetes type 2) than the control group (P < 0.05).

## Discussion

Sexual dysfunction is defined as a person’s inability to engage in the desired sexual intercourse. This disorder can be a symptom of problems with biological origin (biogenic) or intra-psychological or interpersonal conflicts (psychogenic) or a combination of these factors, and any type of stress, emotional disturbances, or lack of knowledge about physiology and sexual function can have a negative effect on the sexual function [63]. Results of various studies revealed that 40% of couples suffer from sexual disorders or relative dissatisfaction with such disorders [64]. Diabetic Individuals are also susceptible to various types of physical and mental disorders and one of the most important neglected care is sexual dysfunction in these patients [65]. Sexual health is an important part of diabetes care, which is particularly neglected in women [66, 67]. Decreased nitric oxide production due to vascular dysfunctions decreases vascular vaginal relaxation and vascular, neurological, and psychiatric disorders are the main causes of reduced desire, vaginal discharge and lubrication, arousal, and orgasm in women with diabetes [67]. Ovarian hormones have an effect on sexual desire; therefore, a factor such as diabetes can disrupt the secretion of these hormones and thus affects the sexual desire of women with diabetes [66]. Diabetes also has a negative effect on the secretion of the endocrine glands at the beginning the vagina and may cause vaginal dryness and irritation, and the couples may experience a painful intercourse [69]; however, another cause of painful intercourse include pelvic, virginal, uterine, and tube infections that may occur more frequently in women with diabetes are [68, 69]; therefore, diabetic women are more likely to experience decreased sexual desire, disrupted ovulation, early menopause, and even infertility than non-infected women [64–69] and these factors showed the importance of control and attention to women with type 2 diabetes.

Nappi et al. reported three critical physiologic requirements, including intact sex steroids, autonomic/somatic nerves, and arterial inflow/perfusion pressure to women’s genital organs play fundamental roles in maintaining women’s sexual function [79] and Maseroli et al. reported Clitoral vascular resistance is positively associated with MetS, decreased sexual arousal, body image concerns, and increased somatized anxiety symptoms [80].

Results of the present study and the review of 3892 individuals aged 18–70 years, the overall prevalence of sexual dysfunction in women with type 2 diabetes was 68.6% based on meta-analysis. Scores of all FSFI (sexual desire, arousal, vaginal lubrication, orgasms, satisfaction, and pain) in diabetic women were lower than non-diabetic women. In order to investigate the effects of potentially effective factors on the heterogeneity of the prevalence of sexual dysfunction in women with type 2 diabetes, meta-regression test was applied to two factors of sample size and year of study, With an increase in the sample size, the overall prevalence of sexual dysfunction is reduced in women with Type 2 diabetes, with an increase in the year of the research, the overall prevalence of sexual dysfunction in women with type 2 diabetes is increased, which was also statistically significant.

Results of a study in the United States reported that 43% of American women aged 18–59 years have sexual concerns [68]; however, Dennerstein et al. [69] reported that 42–88% of women suffer from sexual dysfunction during menstrual cycle. Pontiroli et al., in their systematic review and meta-analysis, reported that the chance for people with type 2 diabetes to develop sexual dysfunction is 2.4% more than that of non-affected people, whereas women with type 2 diabetes are 2 times more likely to have sexual dysfunction than non-affected women [70]. Various studies reported higher prevalence for sexual dysfunction in the diabetic individuals in comparison with the general population, and this factor is considered as one of the most important factors affecting the marital satisfaction in people with sexual dysfunction [71]. Meeking et al. [72] reported a decrease in sexual desire, vaginal lubrication, satisfaction, and orgasm in 64%, 70%, 36%, and 47% of patients, respectively. In another study on patients with type 2 diabetes, Schriener-Engel et al. [73] reported a decrease in sexual desire, vaginal lubrication, and orgasm in patients with type 2 diabetes; while other studies showed the negative impact of type 2 diabetes on sexual desire, sexual satisfaction, lubrication, and orgasm. Enzlin et al. [74] also reports that 35% of women with Type 2 diabetes have orgasmic disorder. Results of this study indicate very high prevalence of sexual dysfunction in women with type 2 diabetes and it is necessary to pay attention to the correlation between sexual function and marital satisfaction. It is also very essential to pay attention to quality of life in women with diabetes so that health policy-makers, physicians, and healthcare providers to place this disorder among the most important and significant disorders in women with type 2 diabetes and to seek out a low prevalence of this disorder through psychological counseling and adopting treatment procedures so as to improve the quality of their marital life and prevent disorders and mental diseases in women.

## Limitations

The most important limitation of the present study is the lack of access to the full text and low quality of some of the studies reviewed.

## Conclusion

Regarding the high prevalence of sexual dysfunction in women with type 2 diabetes, it is essential for health policy-makers to take effective control as well as treatment measures, including periodic sexual care, for women with type 2 diabetes.

## Data Availability

Datasets are available through the corresponding author upon reasonable request.

## Abbreviations

WHO: World Health Organization
FSFI: Female Sexual Function Index
SID: Scientific Information Database
STROBE: Strengthening the Reporting of Observational Studies in Epidemiology for cross-sectional Study
PRISMA: Preferred Reporting Items for Systematic Reviews and Meta-Analysis
